# Evolving roles for the androgen receptor and its protein interactome in castration-resistant prostate cancer

**DOI:** 10.1038/s41388-025-03573-z

**Published:** 2025-09-18

**Authors:** Muj Chukhu, Ujjwal R. Dahiya, Hannelore V. Heemers

**Affiliations:** Department of Cancer Biology, Cleveland Clinic Research, Cleveland Clinic, Cleveland, OH USA

**Keywords:** Prostate cancer, Cancer genetics

## Abstract

The androgen receptor (AR) is a ligand-activated transcription factor that is a major driver of lethal prostate cancer (CaP) progression. Androgen deprivation therapy (ADT) that prevents the binding of androgens to AR has been the mainstay for the treatment of non-organ-confined CaP for more than 8 decades. Although ADT initially induces remissions, eventually resistance occurs while the majority of castration-resistant CaPs (CRPCs) continue to rely on AR’s action for growth. Sustained AR-dependence of CaP that recurs under ADT has historically been linked to AR’s transcriptional activity that controls expression of a distinct program of target genes that mediate aggressive behavior. Recently, less traditional transcriptional roles for AR, such as those impacting non-coding RNAs as well as transcription-independent roles that include AR-dependent splicing programs and translation control have been recognized to contribute to aggressive CaP features and treatment resistance. We reviewed and contrasted the contribution and relevance of these distinct functions for AR during CaP progression. We also considered the roles therein, both overlapping or mutually exclusive, for functionally diverse AR-interacting proteins that have been identified and to date have been mostly considered AR-associated transcriptional regulators. We discuss the potential implications of the involvement of AR interactors in multiple AR-dependent (non-)transcriptional cellular processes for alternative CaP treatment strategies that disrupt AR-coregulator interplay to inhibit AR-dependent transcription when AR ligand-deprivation has failed.

## Introduction: AR continues to drive CaP progression after acquired resistance to ADT

In 2022, prostate cancer (CaP) accounted for an estimated 396,792 fatalities globally [1]. In the United States, CaP remains the second leading cause of cancer-related deaths among men [2]. The treatment options for localized CaP are surgery and radiation, which have the intent to cure the patient [3]. For patients whose CaP recurs after such treatments or who present with CaP that is already too advanced to benefit from these treatments, androgen deprivation therapy (ADT) remains the cornerstone in their treatment plans [3, 4]. ADT inhibits the activity of the Androgen Receptor (AR), a ligand-activated transcription factor that belongs to the steroid hormone group of nuclear receptors and is a major driver of CaP growth and progression. ADT works by interfering with the production of androgens and/or by competing with androgen binding to AR [4].

Already in the 1940s, Charles Huggins and his colleagues demonstrated the effectiveness of ADT through surgical castration, which removes the major source of systemic androgen synthesis, as a treatment for advanced metastatic CaP [5, 6]. Since then, the precise molecular targets and the sequencing and combinations of ADT drugs used have evolved considerably, as has our knowledge of the structural basis that underlies the effectiveness of or resistance to ADT [4]. Historically, first-line systemic ADT mostly took the form of chemical castration through gonadotropin releasing hormone (GnRH) agonists and antagonists, which reduce testosterone levels by suppressing androgen secretion from the testes [4, 7, 8]. To further inhibit the impact of any remaining androgens on AR signaling, antiandrogens, such as bicalutamide were incorporated into the treatment regimens [4, 7, 8]. As discussed in more detail below, it soon became clear that resistance to ADT inevitably develops while CaPs continued to rely on AR action for growth. The recurring CaPs remained AR-positive, even though expression of AR and AR target genes within a patient’s CaP can be heterogeneous [4, 9–13]. Over the next decades, this has prompted several modifications to make ADT increasingly more complete and potent in inhibiting AR, which have been the subject of excellent reviews [4, 7, 8]. Today, second- and higher-generation ADT drugs are administered, often in combination and earlier in CaP progression and more frequently with other classes of CaP drugs, such as the chemotherapeutic docetaxel [14–17]. The therapeutic benefits of high doses of androgens have become clear. These take the form of supraphysiological doses of testosterone that are used in bipolar androgen therapy in which administration of androgen deprivation is alternated with treatment of high doses of androgens [18–20]. These seemingly counter-intuitive approaches of depleting or overdosing androgens were based on the long-recognized biphasic response of CaP cells to androgens in which low doses stimulate cell proliferation and high doses inhibit growth while inducing differentiation [21–23]. Most recently, attention has shifted to proteosomal degradation of AR, with AR-targeting PROTACs being tested as a novel therapeutic option, and with drugs that possess a dual action as AR degrader and as AR antagonist under development [24, 25].

A common theme among all current clinically used ADT approaches is that, despite an initial remission, resistance invariably develops over time [4]. Such acquired resistance to ADT is the major cause for the more than 35,000 CaP deaths in the United States every year [2]. It results, for the most part, from alterations in the AR signaling axis that allow CaP cells to sustain AR activity even under ADT [4, 26, 27]. The exception is a subset of less than 20% of lethal CaPs in which sequential potent ADT induces AR-independence, which are often referred to as neuroendocrine CaP (NEPC) [28, 29].

**Fig. 1:**
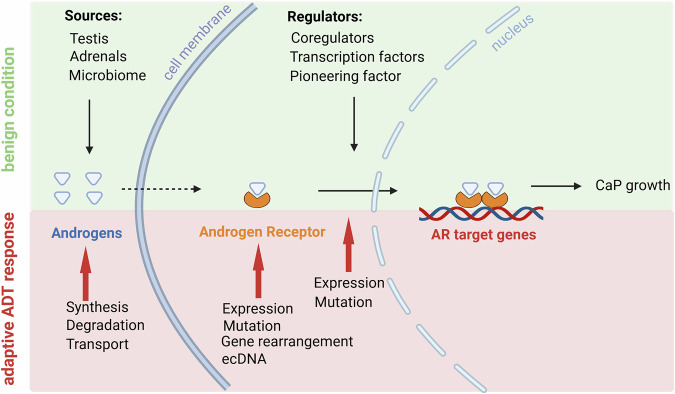
Basic mechanism of AR action. Top half (green) represents the mechanism of AR action before resistance to ADT has developed. Androgens, which can be derived from multiple sources, bind to AR and activate AR. Activated AR relocates to the nucleus where it binds as a dimer to AR target genes and alters their expression with the assistance of coregulators, transcription factors and pioneering factors. Bottom half (pink) indicates the alterations that occur at the level of androgens, AR, and its associated protein interactome to facilitate CaP growth after acquired resistance to ADT. The figure was generated using Biorender.

In its simplest form, the mechanism of AR action entails that androgen binding activates AR to execute a specific transcriptional program that controls CaP growth and progression (Fig. 1) [4, 30]. The pressure of ADT leads to AR-activating alterations in androgen levels, the AR gene, and the elaborate and diverse molecular machinery that AR needs to engage to alter transcription of its target genes. First, even when the testicular synthesis of androgens is blocked, intraCaP androgen levels that are sufficient to activate AR can be achieved. This results from the conversion of androgen precursors produced by the adrenals or the prostate (cancer) microbiome into the mature and most active androgen dihydrotestosterone (DHT) or from intracrine androgen synthesis from cholesterol [4, 31–33]. This can occur because ADT elicits marked changes in the expression or mutation status of several enzymes in the androgen biosynthesis pathways that have been implicated in CaP progression [31, 34]. Dependent on the form of ADT used, build-up of steroid precursors occurs that are shunted to the synthesis of other steroids, such as progesterone (e.g., treatment with CYP17A1 inhibitor abiraterone), some of which can stimulate CaP progression via co-occurring accommodating changes in AR [35]. Indeed, ADT also directly impacts AR’s gene expression, gene arrangement and structural integrity. Androgen depletion causes AR gene and/or enhancer amplification, which renders AR more sensitive to lower levels of ligands, and induces somatic mutations that broaden AR’s ligand-binding spectrum or turn antagonists into agonists [4, 26, 27]. More recently, gene rearrangements have been reported that give rise to AR versions that lack a functional ligand-binding domain (so-called AR variants (ARvs), making them unresponsive to ADT and constitutively active even in the absence of androgens [36]. Other mechanisms that sustain AR action under ADT include expression of AR from ecDNAs or induction of the structurally related nuclear receptor glucocorticoid receptor (GR) that can take over control over at least a fraction of the AR-dependent cistrome and transcriptome [37–39]. Finally, at the third level of AR regulation, ADT dysregulates the expression of and, less frequently, causes somatic mutations in AR-associated transcriptional regulators that, in a context-dependent manner, control the transcriptional output by AR, and thus AR’s effects on CaP cell behavior [30, 40]. These adjustments at three levels of the AR signaling axis, alone or in combination, allow CaP to progress in an AR-dependent manner under ADT. To date it has been impossible to predict the duration of the initial response and remission under ADT and, thus, to adjust treatment plans based on CaP tissue or circulating tumor cell characteristics to maximize such responses. Not surprisingly, among the predictive biomarkers and assays under development, a majority focusses on measuring AR activity and consist of transcriptomics signatures or genomics tools to measure AR’s transcriptional output (e.g., ADT-RS score [41]) or AR’s structural integrity (e.g., AR-ctDETECT [42]). None have yet transitioned to clinical practice or have been incorporated into clinical guidelines.

## Traditional mechanism of AR action

### Basic mechanism of AR action

**Fig. 2:**
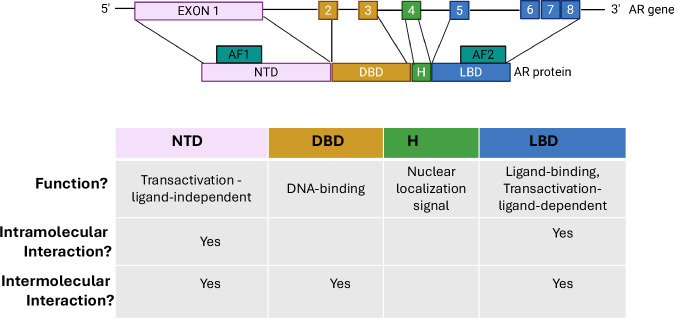
AR domain structure. Top panel: AR domain structure. The scheme shows how the exon organization of the AR gene is translated into AR protein domains. AF, activation function; NTD, N-terminal domain; DBD, DNA-binding domain; H, hinge; LBD, ligand-binding domains. Bottom panel: functions that have been attributed the AR protein domains. The figure was generated using Biorender.

**Fig. 3:**
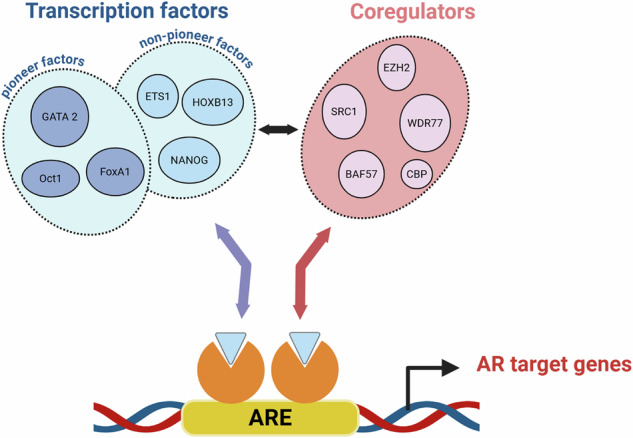
AR interacts with genomic binding sites and with a large protein interactome to alter expression of AR target genes. Androgen-activated AR binds as a dimer with cognate DNA binding motifs that are known as Androgen Response Elements (AREs). The ability of ARE-bound AR to activate or repress transcription of its target genes relies on cooperation with three classes of proteins: pioneering transcription factors, other transcription factors and coregulators. Hundreds of such proteins have been identified. A few representative examples of each of these classes of proteins are named in the figure. The figure was generated using Biorender.

In this review, we focus on insights into the last step of the AR signaling pathway, namely how an activated AR executes its effects on CaP via transcription of its target genes, and how this knowledge has evolved over the past decade. AR shares its modular structure with other members of the nuclear receptor family [43, 44] (Fig. 2). Its highly structurally disordered N-terminal domain (NTD) contains a transactivation function that is constitutively active even in the absence of androgens and whose activity becomes more critical during CaP progression [45]. The DNA-binding domain (DBD) interacts with specific DNA motifs, which are commonly referred to as Androgen Response Elements (AREs), at AR target genes. Via a short hinge region, the DBD is connected to the Ligand-Binding Domain (LBD) to which androgenic ligands and AR antagonists bind and that contains a ligand-dependent transactivation domain [46, 47]. The current view is that AR acts as a dimer, via intra- and intermolecular contacts between monomers that are now recognized to involve most of AR’s domains; NTD, DBD and LBD [48]. This model entails that, upon androgen binding, AR is activated, loses interactions with chaperone proteins, translocates to the nucleus where it binds as a dimer to AREs and activates or represses transcription of its target genes with the assistance of a large interactome of regulatory proteins (Fig. 3). Despite this simple overall mechanism, increasing evidence indicates considerable molecular diversity in the manner in which AR executes transcription, leading to finetuning of and context-dependent output from the AR’s transcriptional complex [30].

### Determinants of heterogeneity in AR’s transcriptional output

#### AR-binding DNA motifs

**Fig. 4:**
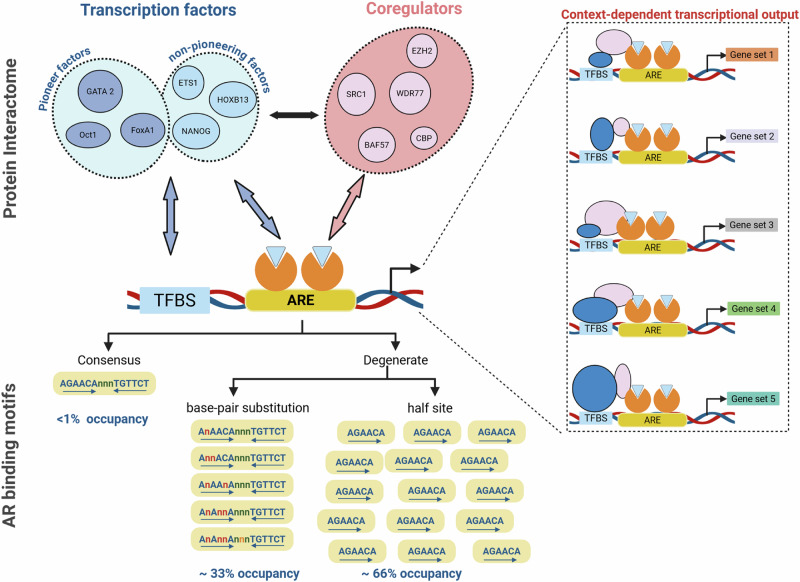
Heterogeneity in AR’s transcription output is determined by context-dependent interactions with its protein interactome and by ARE characteristics. Interactions between AR and its interacting proteins control context-dependent transcriptional output, in which diverse combinations of AR-coregulator-transcription factor interactions regulate the expression of subsets of AR target genes. Coregulators typically do not bind DNA, while transcription factors can be recruited to AREs by protein-protein interactions with AR or by binding to their own cognate transcription factor binding site (TFBS) in close proximity to AREs. Hundreds of AR-interacting proteins have been identified. A few representative examples of each class of AR-associated proteins are named in the figure. The coregulators that are shown represent coregulators that undergo overexpression during the progression from ADT-naïve CaP to CRPC. Less than 1 percent of AREs in CaP cells fit the canonical consensus sequence of two hexamers that are separated by three basepairs. The majority of AREs in CaP cells are degenerate AREs, which have either undergone basepair substitutions in a hexamer or in the basepairs spacing them or consist of a half site (one hexamer only). Two thirds of degenerate AREs are half site AREs. The figure was generated using Biorender.

Most of the reported heterogeneity has been linked to the spectrum of AREs to which AR binds and to the protein interactome that the ARE-bound AR engages, or the chromatome, to alter expression patterns of its target genes (Fig. 4). Canonical AREs consist of a repeat of a 5’TGTTCT3’ hexamer, separated by three base pairs, which resembles the DNA recognition motifs for other NRs, such as GR [49]. Traditionally, the two hexamer half-sites were thought to be organized as palindromic inverted repeats within the 15 bp ARE. None-the-less, already for some of the first AREs that were identified via candidate approaches it was suggested that direct repeats of the two hexamers could occur also [50]. Although this view was later attenuated based on the head-to-head conformation of the DNA-bound DBD dimer that was observed in X-ray crystallography studies and observations of relaxed response element stringency for selective chromatin binding [51, 52], it did point to the potential of variability in the composition of AREs and to deviations from the ideal 15 bp sequence. Applying techniques such as ChIP-chip, ChIP-Seq, ChIP-exo and CUT&RUN, and bioinformatics tools, the work of multiple groups has defined the genome-wide landscape of AR binding sites in CaP cell lines and tissues, which has allowed to more closely examine and characterize the corresponding DNA sequences [53]. Well over 10,000 AR binding sites have been identified in CaP cells, and it has become clear that most AR target genes harbor more than 1 ARE. The majority of AR binding sites (ARBSs) are found in enhancer and intergenic regions rather than in gene promoters, and long-distance interactions occur via chromatin looping between AR bound at AREs at different regions (enhancers versus promoters) of the same AR target gene [53]. The binding of AR to enhancers does not substantially rewire chromatin loops but increases the contact frequency of existing loops with the promoters they interact with [54]. The impact of these interactions on AR target gene expression is proportional to the enhancer-promoter contact frequency. Whether and how all these AREs contribute to target gene transcription and the manner in which they do so at individual target genes still remains to be fully resolved. A recent self-transcribing active regulatory region sequencing (STARR-Seq) study identified 12% of AR binding sites at enhancers as constitutively active, 7% as AR-inducible or AR-activated and the remaining 81% as inactive [55]. Today, the molecular determinants that control ARE (in)activity remain elusive.

Consistent with earlier suggestions, the omics studies that defined and characterized the genomic ARBSs did, however, unambiguously demonstrate that significant degeneracy occurs in the ARE sequence [30, 56] (Fig. 4). Heterogeneity is seen both in terms of the number of basepair mismatches within the textbook 2 hexamer-containing ARE sequence, the number of hexamers per ARE, and the make-up of the spacing between the individual hexamers in AREs. Sequence degeneracy involves mismatches ranging from 1 to 3 base pairs per 15-mer ARE, a higher GC content in the 3 bp spacer between hexamers in imperfect AREs, and over two-thirds of AREs are recognized to consist of half sites only, with a minority making up canonical 15mer AREs [53, 56]. At least 99% of AREs have now been proposed to be degenerate, with degenerate motifs associated negatively with transcriptional outcome and half sites more strongly influenced by transcription factors acting through adjacent motifs or cooperating factors [56].

Divergences from the consensus composition of an AR DNA-binding motif appeared to be associated also with the stage of CaP progression, as changes in ARE composition and location are seen both during prostate carcinogenesis and the development of ADT resistance. Comparing genomic AR binding patterns between benign prostate and localized primary CaP tissues, significant shifts were observed in malignant tissues where new “malignant” AR binding sites were found while “benign” AR sites were lost [57]. The majority of malignant AREs consisted of half sites only. Closer examination of the sequences matching to AR binding peaks revealed enrichment for binding sites for AR-cooperating transcription factors, such as FOXA1 and HOXB13 near AREs. The causal role for these factors in installing the malignant AR cistrome was supported by a benign to malignant shift in AR binding site patterns upon overexpression of these transcription factors in an immortalized benign prostate cell line [57]. These results suggested that cooperation between AR and these transcription factors helps shape and define the AR cistrome. Remarkably, another shift in the AR cistrome was observed between ADT-naive primary CaP and CRPC, with another increase in AR binding sites after ADT resistance [58, 59]. These alterations may be mediated by changes in AR expression and function that emerge during CRPC progression. For instance, overexpression of AR, such as that caused by AR gene amplification or enhancer repetition, increases the number of AR binding sites [60] and genome-wide chromatin relaxation [61]. Similarly, AR variants that result from AR gene re-arrangements or alternative splicing under ADT lead to a novel AR cistrome that at least partially overlaps with that bound by full-length AR [62–64]. In a PDX study of supraphysiologic testosterone treatment, differences in the AR cistrome before treatment could distinguish between responding and non-responding CRPCs. These results suggested that responsive CRPCs exhibit a distinct AR cistrome and differential biological role of the AR compared to CRPCs with innate resistance that could be exploited for the development of a predictive biomarker [65]. Whether the number of canonical, degenerate and/or half-site AREs differs substantially between ADT-naïve CaP and CRPC is less clear. A significant portion of half sites is seen in CRPC as well, and enrichment for binding sites for transcription factors, such as HOXB13 is sustained in the cistromes for full-length AR and for ARvs in CRPC [63]. Given the expansion of the AR cistrome and the emergence of novel AR binding sites under ADT, similar to observations during carcinogenesis, it is tempting to speculate that other factors acting at or near AREs may be in play in CRPC. This possibility is supported by enrichment for binding sites for E2F, MYC/MAX and STATs near AREs, which is reflected in higher activity of E2F3, IL6, and Myc signaling pathways in CRPC compared to ADT-naïve tissues [58]. These results demonstrate another shift in the molecular regulation and output from AR after ADT failure, and suggest that specific targeting of a fraction of AR action by interfering with its interaction with its protein interactome may be valuable.

Most literature to date has focused on the number and location of AR peaks and the type of associated binding motifs that are gained or enriched during CaP progression. This has limited insights into potential functional differences between canonical AREs and those that emerge with increasing disease aggressiveness. In contrast, a recent paper focused on AR binding sites that are increasingly lost as CaP advances, i.e., the canonical 15 mer AREs [66]. The authors performed ChIP-Seq using expression constructs that yield AR DBD domains that are linked to functional domains that convey transcriptional activation or repression, both in benign and malignant prostate cells and organoids. These studies have shown that activating canonical AREs in CaP cells leads to transcription of genes that mediate differentiation and growth suppression. Transcription driven by canonical AREs drives normal prostate lineage differentiation, which, when eliminated, was tolerated by CaP cells but deleterious to normal prostate epithelial cells. The gene signature that results from canonical ARE activity correlated with improved prognosis. These findings suggested that context-dependent transcription from different types of AREs imparts differential aggressiveness to CaP cell behavior, and by extension, that restoring transcription from canonical AREs may provide therapeutic opportunities. Identifying the key molecular determinants that regulate transcription from 15mer AREs, or others, will be critical to such approaches. In this respect, the latter study found that the AR-associated coregulator HDAC3 binds to canonical AREs and modulates growth-suppressive transcription from these motifs. Conversely, loss of HDAC3 increased ARE activation activity from these sites and diminished cell cycle activity, suggesting that HDAC3 inhibitors may hold promise as a CaP treatment to restore an AR cistrome with more benign features [66]. Context-dependent functional differences among AREs fit with findings from another group that combined high-throughput promoter-dependent drug screens and transcriptomics analyses to uncover that low doses of the chemotherapeutic doxorubicin downregulate cell cycle genes while high doses upregulate DNA damage response genes. Mechanistically, low doses increased AR binding to half-site-containing enhancers, whereas AR was lost from canonical AREs. For a subset of genes, AR binding by low doses of doxorubicin increased at sites that harbored binding motifs for prostate-specific factors, such as FOXA1, supporting that AR dependence on cofactors at half sites may be the basis for differential modulation by doxorubicin [67].

#### AR protein interactome

These findings and those showing the involvement of AR-associated transcription factors in establishing a malignant AR cistrome and executing its impact on CaP cell behavior highlight the important role for AR-interacting proteins in the transcriptional regulation of ARE-controlled genes. Hundreds of such proteins have been reported [46, 68]. Apart from transcription factors recognizing binding sites close to AREs and non-DNA interacting coregulators, this interactome also harbors pioneering transcription factors, such as FOXA, OCT and GATA family members that facilitate chromatin accessibility so that AR can bind at AREs. Pioneering factors have been implicated in shaping the AR cistromes, even though several also influence transcriptional output once AR is ARE-bound [30]. While FOXA1’s role as a pioneering transcription factor for AR may stimulate CaP progression, it should be noted that FOXA1 can also restrict AR binding at genes that promote CaP growth, indicating a dual role [69]. Moreover, during CRPC progression, FOXA1 frequently undergoes somatic alterations, some of which can advance the progression of CaP and activate a CaP-specific AR-driven oncogenic transcriptional program [70, 71]. Other specific classes of FOXA1 mutations again differentially impact CaP behavior and outcome [71–73]. AR-associated coregulator proteins show remarkable functional diversity and influence AR transcriptional action in different ways [46]. As expected, this includes facilitating chromatin accessibility, for instance by SWI-SNF proteins, and histone modifications that contribute to this, for instance those exerted by histone acetylases and deacetylases, such as p300 and HDAC3, respectively. On the other hand, the coregulator HIP1 represents a class of proteins that may be less obvious to modulate transcription, namely endocytotic and intracellular protein transporters. Yet others, including SUMO2 and SUMO3, deposit posttranslational modifications on AR and its associated chromatome that control the composition and activity of AR transcriptional complexes. SUMO2 and SUMO3 interacted and modified several AR-associated coregulators (p300, SRC2, BAF proteins) and pioneering factors (FOXA1, HOXB13) associated with ARE-bound AR, demonstrating that SUMOylation amplified interactions with its transcriptional complex. A striking selectivity for AR binding sites based on the presence of adjacent binding motifs emerged in studies in which SUMO2 and 3 activity was inhibited pharmacologically: SUMOylation inhibition using ML-792 inhibited AR binding to genomic regions that contain multiple AREs, but enhanced AR binding to less accessible AREs that are enriched for pioneering transcription factor binding sites. These divergent effects on ARE-bindings were reflected in the expression of AR-dependent genes that were impacted by SUMOylation: genes activated by AR negatively regulate cell proliferation, and those repressed by AR are involved in pattern formation by WNT, TGF-B, and FGF signaling [74].

These results support critical roles for coregulators, and their interactions with other AR-recruited proteins in shaping the AR cistrome and mediating its transcriptional output (Fig. 4). This is consistent with previous of reports of context-dependent action of coregulators at AR target genes, where they preferentially interact with other components of the AR transcriptional complex to control aspects of cell biology and stages of CaP progression [75, 76]. Such activity also fits with allosteric regulation of nuclear receptor activity [77, 78]. Other reports have shown that a single AR-interacting transcription factor, such as HOXB13, can influence AR transcription via different mechanisms. The latter includes interaction with the AR DBD and thereby inhibiting the transcription of ARE-driven genes, the AR-HOXB13 complex conferring androgen responsiveness to genes that harbor a HOXB13-response element, and synergy with AR to enhance the transcription of genes that contain a HOX element adjacent to an ARE [79]. It is clear that AR’s interactions with its protein network, as well as the type of ARE that it binds, can markedly influence the transcriptional output from DNA-bound AR, and, thus, a one-size-fits-all mechanism by which AR regulates transcription from ARE-containing genes does not apply. However, we still lack sufficient understanding of the kinetics and AR interaction modes by which coregulators and cofactors are recruited at specific AREs, the duration of their residence there and the dynamics and hierarchy that control the composition of AR transcriptional complexes. Addressing that gap in knowledge may help define therapeutic strategies to selectively disrupt distinct AR action and overcome the acquired resistance to ADT, which focuses on ligand-depriving the AR and silencing all AR action.

### Allosteric regulation of AR’s transcriptional activity

The mechanistic insights reported above are consistent with allosteric regulation of transcription factor action, in which the type of DNA motif to which the factor binds and its protein and ligand interactions influence its conformational plasticity and the availability of tunable multimerization and protein interaction surfaces that control context-dependent and gene-specific transcriptional activity [78, 80]. Allosteric regulation has long been recognized for other NRs [77, 81] and has become more apparent and accepted as a mode of regulation for AR activity over the past decade, based on literature reports as those described above. However, a lack of structural information has prevented the field from gaining more insights into this aspect of AR action. For the most part, the limited information that is available to date on AR’s overall 3D conformation and the contribution of AR interactors to the overall conformation of AR transcriptional complexes has been derived from in vitro X-Ray, crystallography or computational modeling of individual DBD or LBD domains that are associated with small peptides from AR-associated proteins in the presence of a few agonists or antagonists [44, 51, 82–90]. A recent cryoEM study, for the first time, characterized the structure of full-length AR and defined interaction sites between AR NTD and key AR coregulators p300 and SRC3 [48]. Notably, p300 is one of the most frequently studied AR-associated coregulators in CaP. Its expression was upregulated both by ADT [91] and by the chemotherapeutic docetaxel and was sustained in docetaxel-resistant CaPs [92]. Similarly, SRC-3 also has important roles in lethal CaP progression as it was overexpressed in ADT-naïve CaP patient samples and positively correlated with cell proliferation and resistance to apoptosis [93]. Moreover, SRC-3 expression was elevated in CRPC patient samples, where it negatively correlated with recurrence-free survival [94]. These cryoEM studies did not capture dynamic coregulator/cofactor-dependent changes in AR’s binding surface and context-specific AR function that is consistent with the allosteric regulation of NRs by ligands and coregulators [77, 78, 80, 95, 96]. Such insights are critically important to develop peptides, peptidomimetics or small molecules that specifically and effectively disrupt AR-coregulator interactions that control aggressive CaP behavior.

A more recent paper combined the use of cryo-EM and mass spectrometry on a recombinant AR protein that was bound to a canonical ARE [97]. The authors reported three distinct states for DNA-bound AR, which they named entrenched, splayed and divorced architectures, and which reflect conformations with increasing distance between the two AR monomers. Most of this plasticity resulted from positioning of the 2 LBDs, although the DBD dimerization also allowed for confirmational changes in the AR dimer. Distances between the 2 LBDs and DBD each increased from the entrenched over the splayed to the divorced conformation. Using reporter genes whose activity is driven by ARE half sites or by canonical AREs consisting of two such hexamers, the authors found that cooperativity between the LBDs enables transactivation from weaker, imperfect AREs and noted that direct AREs repeats do reinforce but are not required for AR dimerization. Thus, AR dimerization utilized tunable surfaces that are important for interdomain allostery and were found to be particularly important to bind degenerate DNA sequences, providing an explanation for how AREs that deviate considerably from the optimal textbook sequence in CaP still lead to robust transcriptional outputs. These studies also showed that AR target gene expression from this less-than-optimal cistrome is reinforced by the AR-associated transcription factor ERG. Indeed, the activity of the divorced AR conformational state was more amenable to modulation by ERG. Moreover, using a range of DHT concentrations that partially to fully prime the AR for activity, partially saturated or antagonist-bound AR were most susceptible to regulation by ERG on AR half sites [97]. It will be important to determine to what extent these effects of ERG, as a representative AR-interacting transcription factor, can be extrapolated to other members of the AR protein interactome and how individual interactors impact AR’s allostery.

### AR phase separation

Despite these important new insights, because of the notoriously highly disordered structure of the AR NTD, the latter study used NTD-truncated AR to derive structural information, which may have limited the scope and the therapeutic potential of this information. CaP cells depend strongly on the AR NTD’s ligand-independent transactivation domain AF1, which mediates also transcriptional activity of agonist- and antagonist-indifferent AR variants after acquired resistance to ADT, but to date cannot be effectively inhibited in clinic [45].

Highly disordered proteins or proteins that are characterized by highly disordered regions, such as AR but also several of its interactors, including MED1 and SRC-3 [98], often exhibit liquid-liquid phase separation in membrane-less molecular condensates. Several reports agree that AR exists in such condensates, which fits with the long-recognized punctuate AR nuclear expression patterns, and some reports this holds true also for AR variants [99, 100]. However, the precise AR domain(s) that are most relevant for this phase separation is still being debated [98, 101, 102]. The variability that has been reported for the relevance of the AR NTD, DBD and LBD therein could be due to technical differences in the assays that were used, with some groups performing solely biochemical assays and recombinant AR domain(s) and others combining these with cell line studies. Notwithstanding the reported differences, this cellular distribution characteristic may lend itself to alternative strategies to interfere with AR action, also in CRPC cells. For instance, cysteine-selective irreversible covalent inhibitors reversed AR condensation patterns, led to chromatin condensation and dissociation of AR(v) cistromes and rendered ARv transcriptionally incompetent [101]. These compounds also delayed CaP progression after acquired resistance to ADT, suggesting a potential novel therapeutic strategy to inhibit AR activity.

## Non-traditional mechanisms of AR action

### Processing of AR target genes and transcripts

**Fig. 5:**
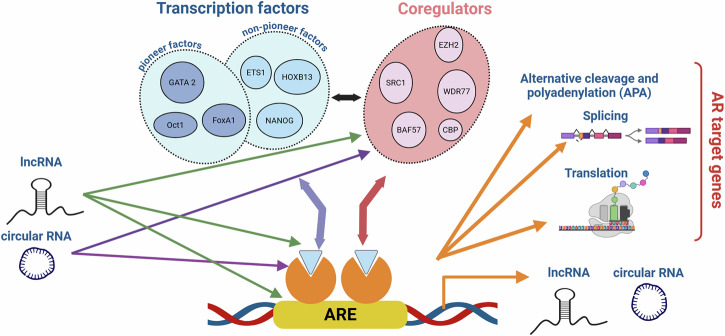
Diverse (post)transcriptional mechanisms further enhance the AR target gene transcriptome heterogeinity. Post-transcriptional mechanisms by which AR diversifies the AR target gene transcriptome include alternative cleavage and polyadenylation (APA), alternative splicing and translation. In addition, AR controls expression of lncRNAs and circRNAs. Furthermore, a growing number of lncRNAs and circRNAs can affect AR expression levels, alter the expression of AR-associated transcriptional regulators or influence transcription of AR target genes. The figure was generated using Biorender.

These renewed insights support the ever-increasing complexity and context-dependency that is being recognized in the control of AR over ARE-driven gene expression, which it executes in large part via interactions with its elaborate and diverse protein interactome. As mentioned, the selectivity that these interactions impart over fractions of AR’s transcriptional output may provide important novel opportunities for alternative therapeutic strategies that disrupt the composition of the select AR transcriptional complexes. In this respect, it is important to consider that the molecular granularity that impacts transcription of AR target genes and is recognized today is likely still an underestimation (Fig. 5). For instance, AR was recently shown to mediate also alternative cleavage and polyadenylation (APA) of AR target genes [103]. The resulting transcriptome reprogramming is an adaptive response of CaP cells to ADT, which alters the composition, and thereby the activity, of protein complexes that regulate APA. Furthermore, several groups have reported that AR controls a spectrum of alternative splicing events that is independent of its traditional role in transcription of AR target genes, allowing AR to further finetune the CaP cell transcriptome [104, 105]. In another post-transcriptional mechanism, AR and androgen deprivation hijack the translation machinery to shape the cellular proteome [106]. This occurs through direct transcriptional control by AR of the translation inhibitor 4EBP1, which AR negatively and ADT positively regulates, respectively.

### Role of non-coding RNAs

These non-traditional roles for AR likely just reflect the tip of the iceberg as it is becoming increasingly obvious also that non-coding RNAs (ncRNAs) have an often-underappreciated role in AR action or are subject to AR control. The recognition that the expression of protein-coding genes can be influenced by non-coding RNAs led to the competing endogenous RNA framework. Initially, the non-coding compartment in this model consisted of microRNAs (miRNAs), small (21-23 nucleotide) single-stranded non-coding RNA molecules that play a crucial role in regulating gene expression by binding to mRNA and preventing its translation into proteins. Competing endogenous RNAs (ceRNAs) regulate other RNA transcripts by competing for shared miRNAs. Since its original formulation, it has become clear that ceRNA does not just involve coding mRNA versus miRNA, but can also be expanded to miRNA binding to long-noncoding RNAs (lncRNAs) and circular RNAs (circRNAs) [107]. The result is an intricate interaction network between different RNA species that act as sponges for the same miRNAs and whose expression levels are determined by competition for shared miRNAs, which constitutes another form of post-transcriptional regulation of gene expression.

Differential expression of miRNAs between benign prostate and CaP and between treatment-naïve localized CaP and treatment-resistant CRPC and NEPC has been reported [108]. Several of these miRNAs target mRNAs encoding AR, AR splice variants or components of the AR protein interactome [109, 110]. Notable examples include miR-99b, which alters expression of AR, the AR-associated coregulator SMARCD1 [111] and the AR target gene PSA [108, 112]. Others, such as miR-205, which is frequently downregulated with miR-200 in cancers, including CaP (e.g., [113]), similarly impact AR expression and are downregulated in CaP, leading to expression of its targets to go up [108, 114, 115]. However, the impact of miRNAs on the development of resistance to both ADT and chemotherapy is much broader and involves a variety of targets and mechanisms [108, 110].

#### lncRNAs

Over the past decade, several studies have made clear that such influence over AR action is not restricted to miRNAs, but that other non-coding RNA species are also differentially involved in prostate carcinogenesis and CaP progression and control or are controlled by AR. Two RNA species that have attracted attention in this regard are lncRNAs and circRNAs.

lncRNAs are generally longer than 200 nucleotides and are mostly not translated into proteins [116]. As for miRNAs, deregulated lncRNA expression occurs during CaP progression. Gene expression profiling efforts have shown that several lncRNAs are upregulated in CaP and during the development of treatment resistance. The increases in their expression can impact AR in a variety of manners, including stabilization of AR mRNA through RNA-RNA hybridization (ARLNC1 [117]), absorption of miRNAs (plncRNA-1 [118]), facilitation of mRNA splicing (PRKAG2-AS1, HOXC-As1 [119]), interference with epigenetic control over AR target genes (SOCS2-AS1 [120]), preventing AR degradation (HOTAIR [121]), regulating its binding to chromatin (PCAT1 [122]) or binding to AR and thereby enhancing its transactivation (PCAT8, PCGEM1 [123]). Fewer lncRNAs that influence AR function are downregulated in CaP. These include, for instance, GAS5, which binds to AR and inhibits its transcriptional activity [124] and LBCS, which binds AR mRNA to inhibit its translation [125].

Apart from starting from differential lncRNA expression, diverse alternative and more comprehensive approaches have been applied to define lncRNAs with relevance to CaP progression and how they impact CaP behavior. Often these strategies have involved mining or generating multi-omics data, and endpoints of interest have included lncRNA’s relevance to CaP prognosis, the molecular function of lncRNAs, or their biological role. One approach was to focus on lncRNAs that are associated with androgen responses by integrating and comparing AR-ChIP-Seq and RNA-Seq data from cells exposed to androgens or antiandrogens and gene expression data from clinical CaPs. This led to the identification of MIR99AHG, DUBR, DRAIC, PVT1, and COLCA1 as lncRNAs with ties to AR signaling [126]. A similar strategy was to focus these efforts on androgen deprivation and consider differential lncRNA expression between benign, ADT-naïve CaP and CRPC tissues. For instance, NAALADL2-AS2 is a novel CRPC-regulated lncRNA whose expression increased under ADT and controlled the expression of genes that regulate cell cycle and glycogen metabolism. NAALADL2-AS2 was proposed to stimulate survival in CaP cells under the pressure of ADT [127]. Other groups have considered specific cell processes, such as anoikis, a form of apoptosis that has been implicated in cancer tumor progression and metastasis and that remains poorly understood in CaP. Following isolation of lncRNAs associated with anoikis-related genes, some were found to predict CaP prognosis, were associated with immune infiltration and susceptibility to fulvestrant, OSI-027, lapatinib, dabrafenib, and palbociclib. The latter results revealed potential therapeutic strategies for CRPC patients with high expression of such lncRNAs [128]. Yet others focused on the RNA species interplay and constructed ceRNA networks based on gene expression data from benign and malignant prostate tissues to identify novel interactors, such as the TRHDE-AS1/hsa-miR-449a/ADAMTS5 axis, which served as a novel prognostic biomarker in CaP [129]. Following the same reasoning, another group manipulated CaP cells to overexpress an miRNA of interest (miR-17-92a), and identified PAINT as a lncRNA that is upregulated in CaP where its expression correlates positively with clinical CaP stages and mediated epithelail-mesenchymal transition [130].

That lncRNA expression itself can be AR-dependent has been recognized [110, 117], providing an indirect way for AR to influence expression of coding or non-coding transcripts, also those that do not necessarily contain an ARE. While lncRNAs do not control the sequence integrity of AREs, lncRNAs such as LINC00844 can regulate global AR binding to chromatin and thus global expression of AR-regulated genes, although the contribution of or preference for specific types of AREs has not yet been established. Notably, because of these features, LINC00844 has been proposed as a coregulator for AR [131]. Expression of LNC00844, a direct AR target, is higher in normal prostate tissues compared to CaP specimens. LNC00844 expression progressively decreases with increasing Gleason scores and in advanced clinical stages [131, 132]. Strikingly, lncRNAs are known to impact the AR protein interactome, the other key determinant of heterogeneity in AR’s transcriptional action. For instance, via RNA-RNA interactions the CaP-overexpressed lncRNA PCLN16 stabilizes mRNA of HIP1, leading to increased expression of this AR-associated coregulator and a positive feedback loop to augment AR signaling [133]. Another lncRNA, SChLAP1, is overexpressed in some CaPs and drives aggressive CaP behavior by promoting metastasis and invasion. It achieves this at least in part by antagonizing the tumor suppressor functions of the SWI/SNF complex [134], several components of which are AR coregulators [46, 68]. SChLAP1 directly interacts with the SWI/SNF factor SNF5 and impairs SNF5-mediated gene expression. The androgen-responsive lncRNA CTBP1-AS is encoded antisense to CTBP1, which is a corepressor for AR. CTBP1-AS is upregulated in CaP and promotes both ADT-naive and CRPC growth. Mechanistically, CTBP1-AS directly represses CTBP1 expression by recruiting the RNA-binding transcriptional repressor PSF along with histone deacetylases [135].

#### CircRNAs

CircRNAs are single-stranded RNAs that form covalently closed continuous loops via back-splicing. Although most are non-coding, some have been reported to encode proteins [136]. Data on circRNAs in CaP progression and AR function are relatively new and still accumulating. Approaches similar to those used to ascertain the CaP relevance for lncRNAs have been employed. Consistent with findings for lncRNAs, circRNAs are differentially expressed during CaP progression. In an isogenic cell line model of enzalutamide resistance, circRNA profiling found over 800 circRNAs, most of which were downregulated in resistant cell lines. One of these repressed circRNAs, hsa_circ_0004870 for which the associated parental genes was RBM39, showed decreased expression also in cells that highly express AR and in malignant cells. This suggests that hsa_circ_0004870 through RBM39, may play a critical role in the development of enzalutamide resistance [137]. The splicing factor RBM39 has since been shown to control alternative splicing of the AR-V7 splice variant from AR pre-mRNA and to control expression of AR-V7 [138]. In another study, circZMIZ1 showed higher expression in the plasma of CaP patients than that of paired benign prostatic hyperplasia (BPH) patients. circZMIZ1 increased the expression of AR and AR-V7 while knockdown of circZMIZ1 inhibited CaP cell proliferation and caused cell cycle arrest at G1 [139]. Another group identified more than 3000 androgen-responsive circRNAs in CaP cells, of which 40% were AR-dependent at the transcriptional level. AR reliance of the remaining circRNAs was suggested to be controlled at the posttranscriptional level, via alternative splicing [140]. In a whole-transcriptome analysis, hsa_circ_0085121 (circRNF19A) exhibited overexpression in CaP cells and samples, and encoded a 490 amino acid polypeptide, which was designated circRNF19A-490aa. Silencing of circRNF19A inhibited proliferation, invasion, migration and docetaxel resistance of CaP cells. Mechanistically, circRNF19A-490aa interacted with HSP90AA1, thereby enhancing AR activity and facilitating activation of Akt/mTOR and PLK1 signaling. circRNF19A-490aa also interacted with HNRNPF, which facilitated recruitment of HNRNPF to the splicing site of AR-V7 and enhanced its alternative splicing. AR is bound to the promoter region of the RNF19A gene, regulating expression of circRNF19A and circRNF19A-490aa [141]. Finally, analyzing an exome-capture RNA-seq dataset from CRPC samples and ribodepletion and RNase R RNA-sequencing of PDX and cell models, circRNAs generated from the AR gene were isolated. Expression of these AR circRNAs was upregulated during castration-resistant progression, with levels of AR circRNAs correlated strongly with those of the linear AR transcripts, indicating a transcriptional mechanism of regulation. These circRNAs were suppressed by androgens and/or AR [142].

## Conclusions, challenges, and limitations

The clinical relevance of the ligand-activated AR for CRPC progression and its value as a therapeutic target has long been evident. Sustained AR reliance of the vast majority of CRPCs has prompted exploring other means of interfering with its function. In this respect, our literature review confirms the attractiveness of AR’s transcription factor moiety and highlights the relevance therein for two of its major determinants, its protein interactome and its DNA binding sites, for context-dependent transcriptional regulation of AR target gene expression. Such heterogeneity may provide opportunities for therapeutic intervention. The latter is evidenced by decreased CaP growth in vitro and in vivo and by inhibition of CaP cell proliferation in explanted tissues after disrupting interactions between AR and its coregulator PELP1 using peptidomimetics [143]. Prevention of AR-protein binding by multivalent peptoid oligomers similarly attenuated CRPC cell growth and overcame enzalutamide resistance [144, 145]. Renewed insights that we reviewed highlighted additional levels of regulation and a more diverse composition for the AR transcriptional complex. For instance, several post-transcriptional mechanisms induced additional variability in the spectrum of AR target gene transcripts. Several were themselves regulated by AR, suggesting that previously unrecognized feedforward and feedback mechanisms exist. This heterogeneity could be achieved by AR’s control over alternative splicing and over translation [104–106]. More recently, non-coding RNAs, such as lncRNAs and circRNAs have also been recognized to influence AR’s transcription factor in diverse manners [110, 142]. These effects have been described mostly for lncRNAs but are starting to emerge also for circRNAs. What fraction of their action is devoted to AR regulation, however, is not clear. None-the-less, their influence includes altering AR expression levels as well as AR’s ability to interact with chromatin at target genes and impacting the expression levels of AR-associated coregulators, such as components of the SWI/SNF complex, HIP1 and CTBP1 [133–135].

How to reconcile these novel contributions with existing insights into the regulation of AR’s transcriptional activity? First, there is a need for a better understanding of whether these roles are direct or indirect. Some lncRNAs have been shown to directly bind chromatin at AR target genes, but whether they have a preference for a specific type of AREs is not clear, nor is the full spectrum of AR target genes’ chromatin environment that is lncRNA-bound. To what extent lncRNAs, or circRNAs, are part of AR transcriptional complexes, and interact with the AR protein interactome is not yet understood. Interaction with the chromatome is a well-recognized mechanism by which lncRNAs can steer gene expression [116]. If and how ncRNAs influence allosteric regulation of AR complexes will need to be resolved. Based on their presence at AR-bound AR target chromatin regions, lncRNAs such as LINC0084 have been proposed to act as coregulator [131], reminiscent of the presence of the SRA RNA in the AR complex [146]. More indirect roles consist of ncRNA-mediated changes in the expression of coregulators, which exist in the cell in limited pools and are involved in the regulation of gene expression by several other transcription factors. How many coregulators are actually subject to similar regulation is not yet clear, but it will be important to ascertain. To what extent ncRNA-mediated changes in their expression influence transcription factor competition for these regulators remains to be determined. Considerable attention will need to be dedicated to higher order structure and stoichiometry of composition of AR complexes that contain both protein and ncRNA transcriptional regulators. It may also be worthwhile to reanalyze gene expression datasets obtained from CaP models in which coregulator function is altered and apply ceRNA analyses to gain insights into cooperating transcript networks.

Future studies will need to examine in more detail also the impact of specific CaP stages and treatments on the contribution of ncRNAs to AR action. Similar to coregulators [40], a significant fraction of ncRNAs appears to be AR- and ADT-controlled [110], so their action could be influenced by ADT or BAT, or by changes in AR expression, AR gene rearrangements or AR mutations that occur under the pressure of ADT. In this respect, CaP treatments could influence the composition of the ligand milieu, which may further impact on AR conformation’s and action as ligand represents the third determinant of NR allostery [77].

These added levels of complexity and the limitations that will need to be overcome to fully understand their implications and implement these insights in targeting AR action for therapeutic intervention could be viewed as challenging. At the same time, they may offer novel strategies as for instance antisense oligos (ASOs), which are already FDA-approved to treat other human disorders [147, 148], are able to disrupt ncRNA interactions in transcriptional complexes [149]. ASOs that target the lncRNAs PCAT6 and LINC01126 have already been shown to be able reduce CaP growth [150, 151]. Preventing the proper composition of AR interactomes at subsets of AR target genes may overcome the acquired resistance to ADT and bypass the cell plasticity that results in the emergence of NEPC.

## Data Availability

Not applicable.
